# A Monoclonal Antibody That Tracks Endospore Formation in the Microsporidium *Nosema bombycis*

**DOI:** 10.1371/journal.pone.0121884

**Published:** 2015-03-26

**Authors:** Yanhong Li, Meiling Tao, Fuping Ma, Guoqing Pan, Zeyang Zhou, Zhengli Wu

**Affiliations:** 1 State Key Laboratory of Silkworm Genome Biology, Southwest University, Chongqing 400715, P.R. China; 2 College of Animal Science and Technology, Southwest University, Chongqing 400715, P.R. China; 3 The Sericultural Research Institute, Jiangsu University of Science and Technology, Zhenjiang, Jiangsu 212018, P.R. China; 4 Laboratory of Animal Biology, Chongqing Normal University, Chongqing 400047, P.R. China; 5 Fisheries Ecology and Environment Laboratory, Southwest University, Chongqing 400715, P.R. China; Institute of Plant Physiology and Ecology, CHINA

## Abstract

*Nosema bombycis*, the first identified microsporidium, is a destructive pathogen of the silkworm *Bombyx mori* and causes severe worldwide economic losses in sericulture. Major microsporidian structural proteins, such as the spore wall protein (SWP), are known to be involved in host invasion. In this study, the reactivity of the monoclonal antibody 2B10 was tested against an endospore protein of *N*. *bombycis* with a molecular weight size at 50-kDa, using Western blotting. The antigen was purified after immunoprecipitation and was further identified as EOB13320 according to MALDI-TOF MS assay. We found that EOB13320 locates to the surface of the different developmental stages of the parasite, mostly the sporoblast stage and the mature spore after immunoelectron microscopy examination. EOB13320 was also widely distributed in the developing endospore, especially at the sporoblast stage. This endospore protein also accumulated in the cytoplasm of both the merogony and sporoblast stages. These results imply that EOB13320 detected by monoclonal antibody 2B10 is expressed throughout the life cycle of the parasite, notably during the stage when the endospore is formed, and that this protein is important for spore-coat formation and parasite maintenance. Our study could be instrumental in the understanding of spore wall formation and will help to gain greater insight into the biology of this parasite.

## Introduction

Microsporidia are obligate intracellular spore-forming parasites that are widely acknowledged as highly reduced and derived and are most closely aligned with fungi [1–5]. There are currently over 1300 described species of microsporidia in more than 160 genera [6] and they infect a wide range of hosts, including insects, fish, birds, and mammals [7–9]. The microsporidium *Nosema bombycis*, which was first identified by Nägely in 1857, is a lethal agent that causes pebrine disease in the silkworm *Bombyx mori*. Like most microsporidia species, all parasitic stages of *N*. *bombycis* are in direct contact with the host cell cytoplasm [10], and the internalized parasite cycle generally includes a meront cell division phase (or merogony), a spore production phase (or sporogony), and a mature spore or infective phase [11,12]. The infective spore is the only stage that happens outside of the host, and the spore can survive for a long time [13]. When a potential host cell lies nearby, the infective spore is triggered, and germination begins. The sporoplasm is injected into the host cell cytoplasm by the extrusion of the polar tube. The intracellular sporoplasm is quickly surrounded by a plasma membrane and develops into a meront. Following merogony, the cells are covered by an outer membrane consisting of electron-dense materials and enter into sporogony [14]; at this point, the parasite is defined as a sporont. The sporonts undergo continuous transition into sporoblasts, whose most distinguishing features are the formation of the early extrusion apparatus (polar tube) and the assembly of the final spore shape. These sporoblasts develop into mature spores, which have polarized organelles and possess thick walls.

The microsporidian spores can survive outside their hosts and retain infectivity for a long period [13] because they have a rigid spore wall that consists of an exospore, an endospore and a plasma membrane. The spore wall protects the mature spore from the environment. Very few studies have characterized the functions and components of the microsporidian spore wall proteins. So far, only eight different proteins from the family *Encephalitozoonidae* have been identified, five exosporal proteins (EcSWP1, EiSWP1, EiSWP2, EcExP1 and EhSWP1) [15–18] and three endosporal proteins (EnP2 or SWP3, EnP1 and *Ec*CDA) [19–22]. In *N*. *bombycis*, six spore wall proteins have been isolated, the exosporal proteins SWP5, SWP26, and SWP32, and the endosporal proteins SWP25, SWP30, and *Nb*SWP12 [23–28]. Wang et al. reported some peptides from *N*. *bombycis* spore wall proteins [29], and several antibodies against spore wall antigens have been raised [30,31]. All of these studies focused on the components of spore wall proteins; however, the functions of these spore wall proteins are still unknown.

In our previous study, we developed two monoclonal antibodies (mAbs) that recognize spore wall proteins of *N*. *bombycis*. Using Western blotting, we showed that one of the two mAbs, mAb 2B10, recognized a 50-kDa protein in a soluble spore protein fraction [30]. The current study focused on the localization and expression of the target antigen of mAb 2B10. Our results indicate that the distribution of EOB13320, the target antigen of mAb 2B10, is dependent on the spore’s developmental stage. EOB13320 is gradually transported from the cytoplasm to the spore wall following spore development, resulting in the presence of this protein in the endospore and its disappearance from the cytoplasm in mature spores. Therefore, we hypothesized that EOB13320 is a critical protein which is associated with the formation of the endospore and the maintenance of *N*. *bombycis*.

## Materials and Methods

### 2.1 Nosema bombycis spore production and purification

*N*. *bombycis* (Chongqing isolate CQ1, CVCC no. 102059) spores were produced and purified as previously described [23]. Spores were propagated in laboratory-reared silkworm larvae and were purified from the infected silk glands of fifth instar larvae by centrifugation over a discontinuous Percoll gradient. The purified spores were washed and stored with antibiotics (100 μg/mL streptomycin, 100 U/mL penicillin, Sigma) at 4°C.

### 2.2 Purification of the monoclonal antibody

The mAb 2B10 was purified from ascites by precipitation with ammonium sulfate and dialysis against PBS (24 h at 4°C) [30] and was separated using 12.5% SDS-PAGE.

### 2.3 Protein extraction and immunoprecipitation

*N*. *bombycis* spores were induced at 28°C with 0.1 M KOH for 30 min, collected by centrifugation (20,000 g, 10 min) and subjected to protein extraction using cell lysis buffer (Beyotime). After centrifugation (20,000 g, 5 min), the supernatant was collected and analyzed using immunoprecipitation (IP), as previously described [27]. A total of 4 μg of mAb 2B10 was added to 100 μL (1 μg/ μL) of the spore supernatant and incubated overnight at 4°C, and then 100 μL Protein A+G Agarose (Sigma) was added and incubated for another 3 h at 4°C. The mixture was centrifuged (22,000 g, 5 min) and washed with cell lysis buffer (Beyotime). The samples were boiled for 5 min and stored for further research.

### 2.4 Gel electrophoresis and Western blotting

Protein samples were characterized by standard 12.5% SDS-PAGE. For image analysis, the gels were visualized using silver staining. Or for immunoblotting studies, the gels were transferred onto polyvinylidene difluoride (PVDF) membranes (Millipore). The protein marker lane was cut and stained with Coomassie Brilliant Blue R-250 (Sigma); the remaining membrane was blocked with a solution of 5% skimmed milk powder in PBS, probed with the primary antibody (1:2,000 dilution of the mAb 2B10 ascites fluid) and a secondary antibody (Mouse TrueBlot ULTRA: Anti-Mouse Ig HRP, 1:2,000 dilution, Rockland Immunochemicals, Gilbertsville, PA), and detected using an ECL system.

### 2.5 MALDI-TOF analysis

The MALDI-TOF MS method was employed to analyze the 50-kDa protein, as previously described [23,25]. Briefly, soluble *N*. *bombycis* proteins were extracted and subjected to immunoprecipitation as described above. IP samples were separated by SDS-PAGE, and the 50-kDa protein band was excised and further analyzed by mass spectrometry using a Voyager DE PRO MALDI-TOF-MS (Applied Biosystems). The GPMAW software (version 6.10, Applied Biosystems) database, which was constructed using the local protein database of *N*. *bombycis* [32], was used for PMF analysis using monoisotopic peptide masses and a peptide mass tolerance of 0.5 Da. Sequence was analyzed using online tools (http://www.cbs.dtu.dk/services/).

### 2.6 Indirect immunofluorescence microscopy (IFA)

An indirect immunofluorescence assay was performed as described in detail previously [25]. Briefly, purified mature spores of *N*. *bombycis* were fixed with 80% cold acetone for 10 min at 4°C. After the spores had dried naturally, the cells were permeabilized with 70% ethanol/0.5% Triton X-100 and blocked with 2% goat serum/5% BSA in PBS buffer, incubated with a 1:400 dilution of either mAb 2B10 or an isotype control (mouse IgG, Sigma) for 1 h at RT, washed, and incubated with fluorescein isothiocyanate (FITC)-conjugated goat anti-mouse IgG (1:64 dilution, Sigma) for 1 h. The nuclei were stained with DAPI. All images were obtained using an Olympus BX50 fluorescence microscope equipped with a 100 × objective lens (excitation wavelength of FITC-IgG: 495 nm; DAPI: 359 nm; magnification, 100×).

### 2.7 Immunolocalization studies

The immunolocalization assay was performed as previously described [23]. Briefly, the silk glands of *N*. *bombycis*-infected fifth instar larvae of silkworms were collected and fixed in 4.0% paraformaldehyde/0.1% glutaraldehyde in 0.1 mol/L sodium cacodylate buffer (pH 7.2) for 2 h at 4°C. The specimens were dehydrated using a sequential series of 30%, 50%, 70%, 90%, and 100% ethanol, imbedded and photopolymerized in K4M resin, and sectioned onto grids. The specimens were blocked in 2% goat serum/5% BSA in phosphate buffer, incubated for 2 h with a 1:200 dilution of either mAb 2B10 or mouse IgG (Sigma), washed, and incubated in 1:100 diluted gold-conjugated anti-mouse IgG (10 nm ID, Sigma) for 1 h. The specimens were observed under a Hitachi H-7500 transmission electron microscope (TEM) at an accelerating voltage of 80 kV.

## Results

### 3.1 Identification of mAb 2B10 and its antigen

**Fig 1:**
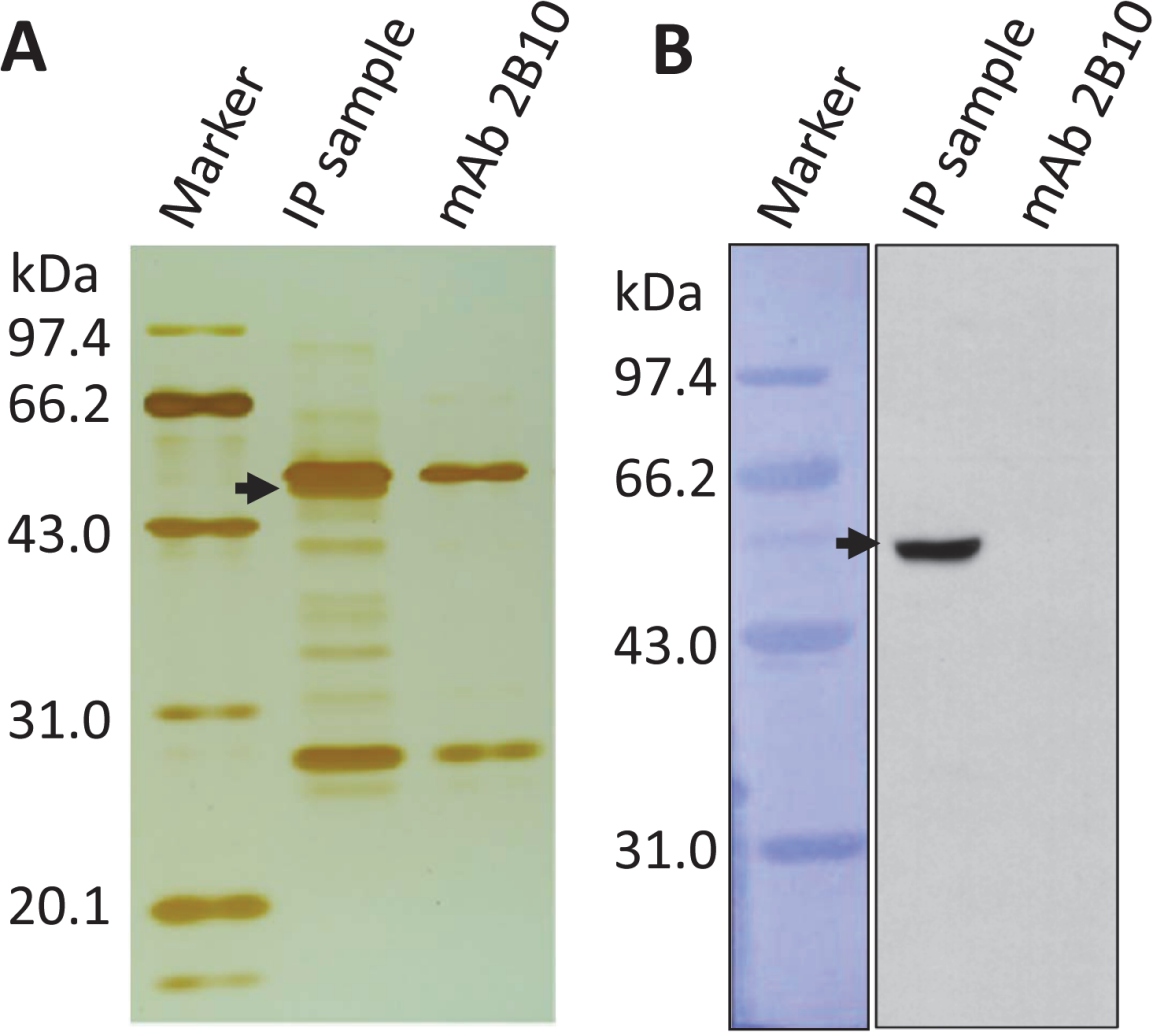
MAb 2B10 and its antigen identified by SDS-PAGE and Western blotting. *N*. *bombycis* spores were purified and lysed, and the lysates were subjected to immunoprecipitation (IP) analysis using mAb 2B10 binding. The IP samples were separated by SDS-PAGE and visualized by silver staining (***A***) or transferred onto PVDF membrane for Western blotting (***B***). MAb 2B10 was run in parallel as a control. The target antigen, with a molecular weight of approximately 50 kDa, is shown with arrows. A representative of *n* = 3 independent experiments is shown.

By SDS-PAGE separated, the monoclonal antibody, 2B10, consisted of a 53-kDa heavy chain and a 23-kDa light chain (Fig 1A), which suggested that mAb 2B10 is an IgG immunoglobulin. To further identification the antigen of mAb 2B10, immunoprecipitation (IP) method, which is useful for the isolation and purification of target proteins from an immune complex, was performed. *N*. *bombycis* lysates were incubated with mAb 2B10, and the antigen-antibody complex was collected using Protein A+G-Agarose and separated using SDS-PAGE. The SDS-PAGE gel was visualized using silver staining (Fig 1A) or transferred onto a PVDF membrane for Western blotting (Fig 1B). To exclude IP antibody contamination, a sample of mAb 2B10 was run in parallel on the gel for silver staining and Western blotting. In addition to the two antibody bands, several other proteins bands were detected (Fig 1A), suggesting that mAb 2B10 maybe recognizes several antigens in *N*. *bombycis*. To exclude non-specific binding, a TrueBlot secondary antibody was used for Western blotting, and, as shown in Fig 1B, only a 50-kDa protein was detected. No bands were detected in the mAb 2B10 only sample lane. These results indicate that mAb 2B10 specifically targets the 50-kDa antigen.

### 3.2 MALDI-TOF MS analysis of the antigen of mAb 2B10

**Fig 2:**
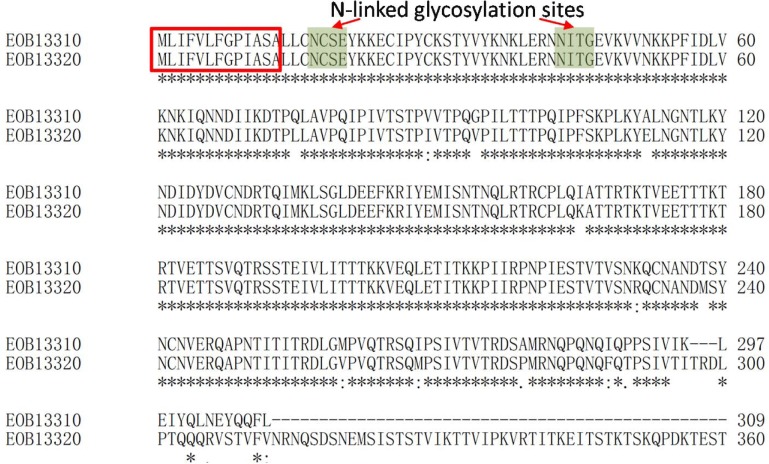
Multiple alignment of hypothetical protein EOB13320 and EOB13310 by Clustal. Identical amino acids are labeled with asterisks, the predicted signal peptide is marked with red rectangular box, and predicted N-linked glycosylation sites are marked with green.

To discover further information about the antigen of mAb 2B10, the IP sample of 2B10 was separated by SDS-PAGE, and the 50-kDa protein band was excised and analyzed by MALDI-TOF MS. Several peptides and their observed molecular weights were obtained (S1 Fig). After searching our local protein database of *N*. *bombycis*, the relative peptide sequences were obtained from two hypothetical proteins (Table 1), GenBank accession No. EOB13320, a 117-kDa protein composed of 1041 aa, and No. EOB13310, a 35-kDa protein composed of 309 aa. Interestingly, the N-terminal domains of EOB13310 (positions 1 to 309) and EOB13320 (positions 1 to 312) are 96% identical at the protein level (Fig 2). Both proteins have a predicted signal peptide (positions 1 to 13), two predicted N-linked glycosylation sites (positions 17 to 20 and 43 to 46), and phosphorylation sites. Since EOB13310 has 35-kDa which is lower than the molecular weight (50-kDa) indicated by Western blotting as shown in Fig 1B, we conclude that the molecule detected by mAb 2B10 was likely a fragment cleaved from EOB13320 at 117-kDa. Currently we are working on the questions why EOB13320 is cleaved and whether the cleaved fragment has novel functions.

**Table 1:** MALDI-TOF MS analysis the 50-kDa protein of *N*. *bombycis*.

**Protein ID**	**pI/MM (kDa)**	**Cov (%)**	**MMobs**	**MMcalc**	**MMdiff**	**Position**	**Sequence**
GenBank ID: EOB13320. hypothetical protein.	10.0/117.06	3.8	1245.485	1245.467	-0.02	159–169	TRCPLQKATTR
			1504.068	1504.553	0.49	120–131	YNDIDYDVCNDR
			1933.964	1934.182	0.11	282–298	NQPQNQIQPPSIVTITR
			2106.014	2106.321	0.31	120–136	YNDIDYDVCNDRTQIMK
GenBank ID: EOB13310. hypothetical protein.	9.3/35.07	12.9	1245.485	1245.467	-0.02	159–169	TRCPLQKATTR
			1504.068	1504.553	0.49	120–131	YNDIDYDVCNDR
			1933.964	1934.182	0.11	282–298	NQPQNQIQPPSIVTITR
			2106.014	2106.321	0.31	120–136	YNDIDYDVCNDRTQIMK

### 3.3 EOB13320 expression in *N*. *bombycis*

**Fig 3:**
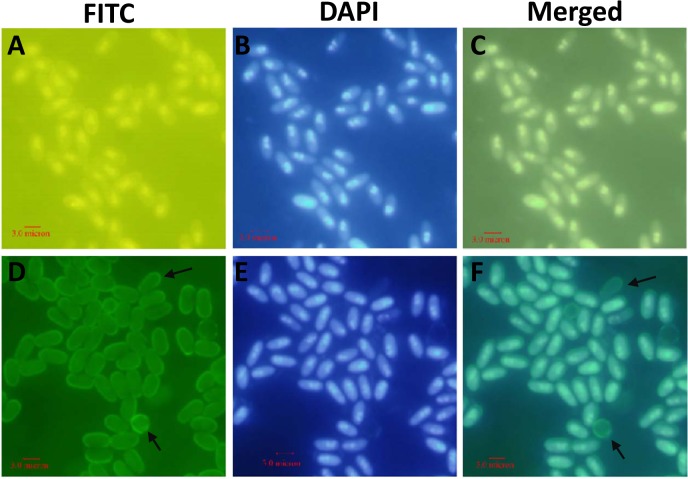
Immunofluorescence labeling of mature spores of *N*. *bombycis*. Spores were permeabilized and detected using mouse IgG (***A***) and mAb 2B10 (***D***). The antigen of mAb 2B10, EOB13320, was located on the spore wall. The arrows indicate the empty spore wall. (***B***) and (***E***), DAPI staining of the same visual field as in (***A***) and (***D***), respectively. (***C***) and (***F***), merged images of (***A***) plus (***B***) and (***D***) plus (***E***), respectively.

To determine the expression of EOB13320 in the mature spores, immunofluorescence assays (IFA) were performed in mature *N*. *bombycis* spores using mAb 2B10. The purified spores were permeabilized with 70% ethanol/0.5% Triton X-100, labeled with mouse IgG (Fig 3A–3C) or mAb 2B10 (Fig 3D–3F), and developed with a FITC-conjugated goat anti-mouse IgG. A signal was detected in the spore coat labeled with mAb 2B10 (Fig 3D), notably in the empty spore coat (marked by black arrows). No signal was detected in the spores labeled with mouse IgG (Fig 3A), or when the spores were not permeabilized (data not shown). These data suggest that EOB13320 is exclusively expressed on the endospore of mature spores but not on the exospore. The nuclei were stained with DAPI.

### 3.4 Ultrastructural localization and developmental expression of EOB13320

**Fig 4:**
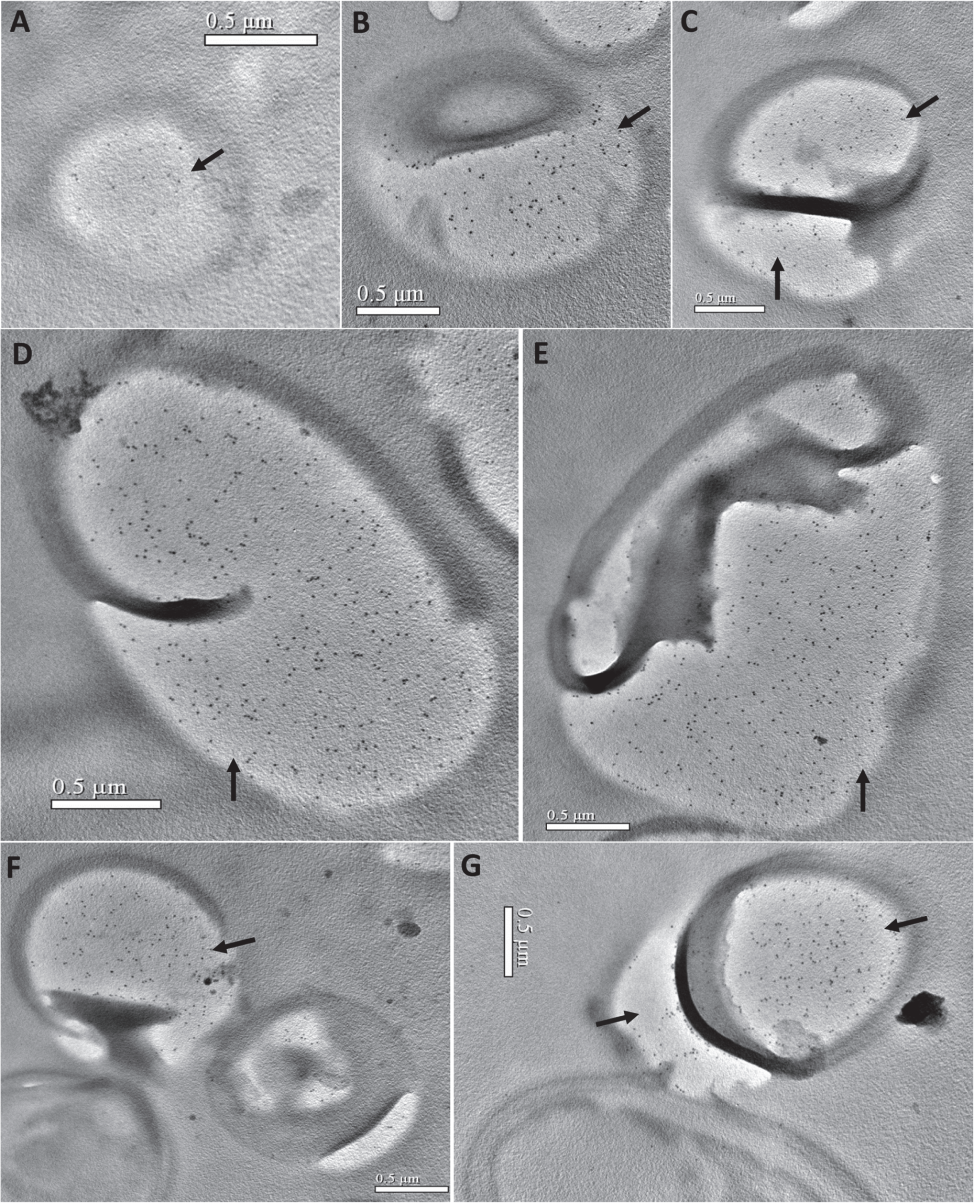
Immunoelectron microscopy of the merogony stage of *N*. *bombycis* in the infected silk glands of silkworms. *N*. *bombycis*-infected silkworm silk glands were isolated, fixed, embedded, ultrathin sectioned, and labeled with mAb 2B10 and 10 nm colloidal gold conjugated-secondary antibody. ***A***, EOB13320 (black arrow) is observed in the sporoplasm of the meronts. ***B-G***, There are numerous gold labels (black arrows) in the sporoplasm of the cells in transition from meront to sporont during merogony. Bars = 0.5 μm.

**Fig 5:**
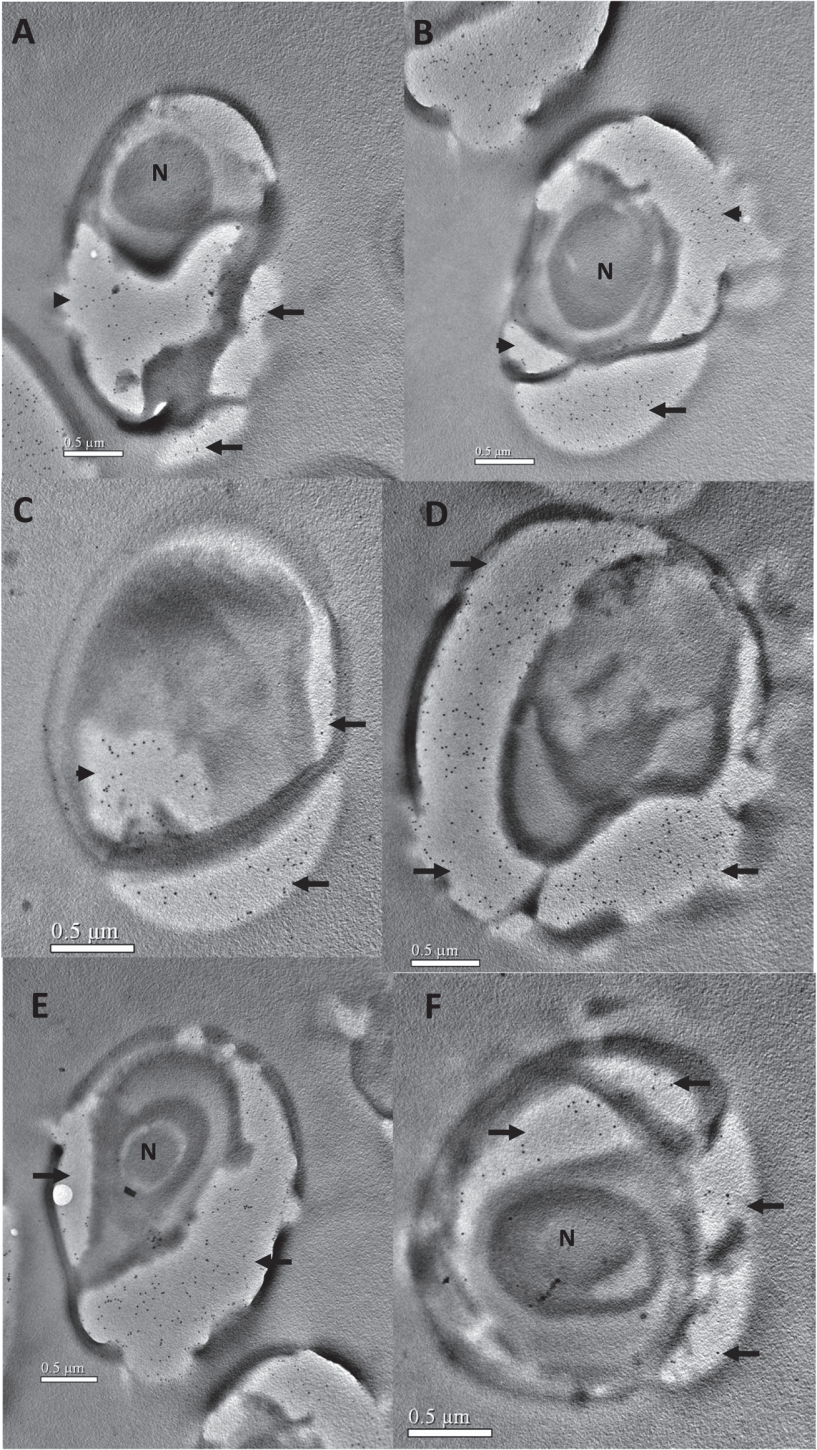
Immunoelectron microscopy of *N*. *bombycis* during sporogony. Samples were prepared and labeled as described in Fig 3. ***A*** and ***B***, The developing sporont. The gold labelling is concentrated in the dividing sporoplasm (arrowheads) and the developing endospore (arrows). ***C-F***, Gold labeling was decreased in the sporoplasm and greatly increased in the developing endospore during sporogony (arrows). N, nucleus. Bars = 0.5 μm.

**Fig 6:**
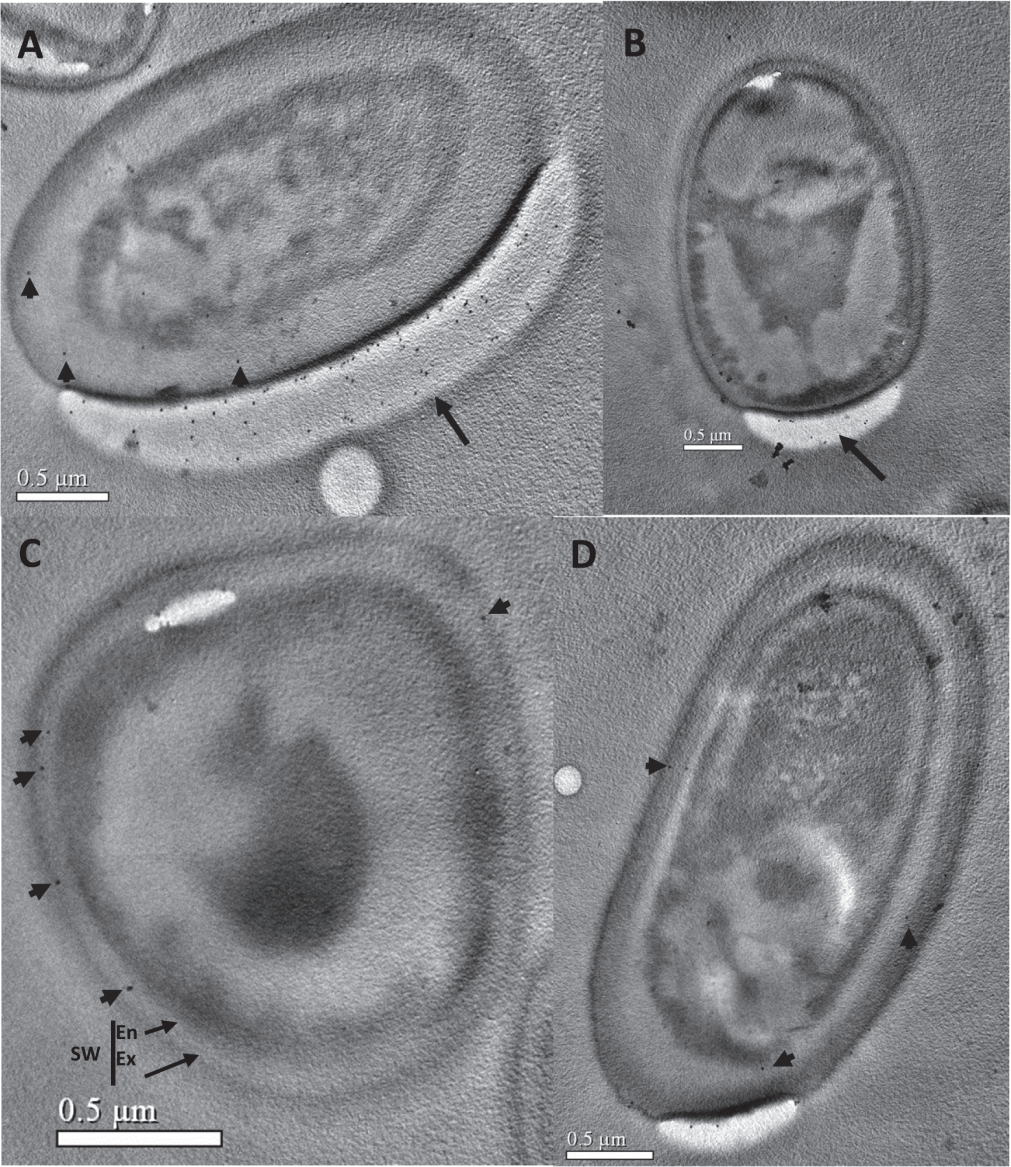
Immunoelectron microscopy of *N*. *bombycis* during the premature and mature stages. Samples were prepared and labeled as described in Fig 3. ***A*** and ***B***, In premature spores, patches of gold particles accumulate in the developing endospore (arrows), with very few labels scattered in the complete endospore (arrowheads). ***C*** and ***D***, In mature spores, some gold labeling is present in the endospore (arrowheads), but not in the sporoplasm or exospore. SW, spore wall; Ex, exospore; En, endospore. Bars = 0.5 μm.

To examine the expression of EOB13320 during the spore’s developmental stages and to further determine its cellular localization, immunoelectron microscopy (IEM) was used. The *N*. *bombycis*-infected silk glands were fixed, embedded, sectioned, and labeled with mAb 2B10. IEM of ultrathin sections demonstrated that EOB13320 was present in various meronts and sporonts, and throughout all stages of endospore development (Fig 4, Fig 5 and Fig 6).

A number of gold particles were present in the sporoplasm of *N*. *bombycis* meronts (Fig 4A) and in particular in the sporoplasm of cells in the transition stage from meront to sporont (i.e., merogony) (Fig 4B–4G). The quantity of gold particle was greatly increased with the thickening of the plasma membrane (Fig 5). During the development of the sporonts, numerous gold particles were present in the sporoplasm (Fig 5A and 5B). When the sporoplasm was completely surrounded by an unequally thickened membrane, many gold particles were distributed on the densest region of the plasma membrane (Fig 5A–5C). In the sporoblasts (Fig 5C–5F), EOB13320 was located on the forming spore wall (marked as arrows). The number of gold particles significantly increased in the spore wall sduring sporogony, and decreased in the sporoplasm. As the spore developed into the premature and mature stage, gold particles were mainly observed in the developing endospore (Fig 6) and developed endospore (arrowheads). Gold particles were consistently present only on the inner electron-lucent layer of the spore wall, but not on the exospore, polar filaments, and other inner structures of the spore.

These results show that the subcellular location of EOB13320 is dependent on the developmental stage of the spore, and that, following spore development, the antigen is gradually transported to the endospore, until it is only present on the endospore of mature spores. The results indicate that EOB13320 not only plays a critical role in the formation of endospores, but is a major protein in *N*. *bombycis* endospore maintenance. No gold particles were found in the spores labeled with mouse IgG (data not shown).

## Discussion

In this study, we report the characterization, reactivity and antigen recognition of the previously developed mAb 2B10 [30] and assess the developmental expression of the target antigen of mAb 2B10, EOB13320, in all the parasitic stages. Immunofluorescence assays ascertained that EOB13320 is associated with the spore wall and is located on the inner surface layer of mature spores of *N*. *bombycis*. However, it is unclear how EOB13320 is tethered to the endospore of mature spores. The immunolocalization of EOB13320 revealed that it is abundantly expressed by cells in the meront-sporont transition stage and is present throughout the intracellular development of the pathogen. During merogony, EOB13320 was observed within the whole cytoplasmic space of the meronts, the transition stage from meronts to sporonts, and the sporonts. EOB13320 density also increased as the cells developed, implying that EOB13320 is important in spore development. Moreover, during sporogony, the accumulation of EOB13320 was targeted to the forming endospore. Notably, during the mature stage, EOB13320 density decreased on the endospore and disappeared in the cytoplasm.

Microsporidian spores are known to possess a thick spore wall, which is composed of an electron-dense, proteinaceous exospore and an electron-lucent endospore containing chitin and protein [11]. This wall confers the rigidity and the resistance to various environmental stresses. Because the microsporidian protein-chitinous cell wall interferes with cell disruption and protein solubilization by different harsh lysis treatments [16,33], the quantity and quality of spore wall component protein extracts is limited, and the sequence similarity between SWPs is low. Thus, although the first microsporidians were discovered more than 150 years ago, studies on structural SWPs are few and basic. In previous studies, few spore wall proteins have been successfully identified. Most of the antibodies directed against microsporidia were specific to spore wall antigens in *Encephalitozoonidae* [34–38] and *N*. *bombycis* [29–31]. There are a few reports on related proteins or antibodies that describe the development process of the parasitic endospore. There is only one study, Brosson D et al (2005), on *Ec*CDA, a surface protein implicated in microsporidian spore wall formation [19]. In the current work, immunoprecipitation was used to successfully isolate the specific antigen targeted by mAb 2B10. The differential expression of EOB13320 at all microsporidial developmental stages was analyzed, which led to the discovery that EOB13320 is an endospore protein and is directly associated with endospore formation. Thus, our studies will not only benefit research on the biosynthesis and organization of the spore wall but may also improve our understanding of the biological developmental process of this microsporidia.

The current study showed that EOB13320 is a novel protein that is different from all known proteins and is very important for endospore development and maintenance. EOB13320 will be a new treatment target for microsporidiosis. Our study also suggests that mAb 2B10 will be useful in immunoprotection studies and for new diagnostic reagents for microsporidiosis caused by *N*. *bombycis*. Further studies will attempt to discover how EOB13320 is cleaved and whether the cleaved fragment has novel functions, because it could play a key role in elucidating the mechanisms of *N*. *bombycis* spore development and the mechanisms underlying host parasite interactions.

## Supporting Information

S1 Fig MALDI-TOF MS spectra of 50 kDa protein. The mAb 2B10 were used to IP from the soluble protein of *Nosema bombycis*. And IP sample were separated by SDS-PAGE, the 50 kDa protein band were excised and digested by trypsin and submitted to MALDI-TOF MS analysis.
(PDF)
