# Supplementary material for: A Monoclonal Antibody That Tracks Endospore Formation in the Microsporidium Nosema bombycis
Source: PLoS One. 2015 Mar 26;10(3):e0121884. doi: 10.1371/journal.pone.0121884 (PMC4374874; doi:10.1371/journal.pone.0121884)

Supplemental Figure 1: MALDI-TOF MS spectra of 50 kDa protein. The mAb 2B10 were used to IP from the soluble protein of *Nosema bombycis*. And IP sample were separated by SDS-PAGE, the 50 kDa protein band were excised and digested by trypsin and submitted to MALDI-TOF MS analysis.

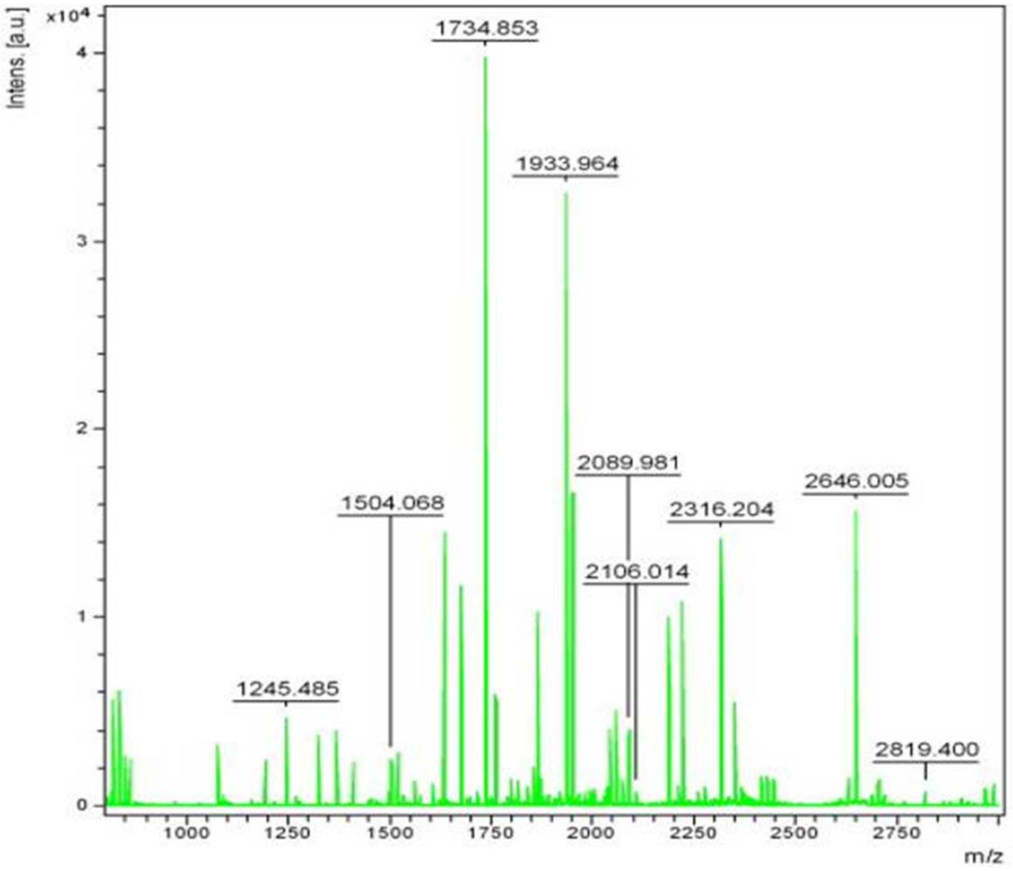

Supplement: S1 Fig — The mAb 2B10 were used to IP from the soluble protein of Nosema bombycis. And IP sample were separated by SDS-PAGE, the 50 kDa protein band were excised and digested by trypsin and submitted to MALDI-TOF MS analysis. (PDF) [file pone.0121884.s001.pdf]
